# Composites prepared from the waterborne polyurethane cationomers—modified graphene. Part I. Synthesis, structure, and physicochemical properties

**DOI:** 10.1007/s00396-014-3417-3

**Published:** 2014-10-26

**Authors:** Piotr Król, Bożena Król, Kinga Pielichowska, Milena Špírková

**Affiliations:** 1 Department of Polymer Science, Faculty of Chemistry, Rzeszów University of Technology, Al. Powstańców Warszawy 6, 35-959 Rzeszów, Poland; 2 Department of Biomaterials, Faculty of Materials Science and Ceramics, AGH University of Science and Technology, Al. Mickiewicza 30, 30-059 Kraków, Poland; 3 Nanostructured Polymers and Composites Department, Institute of Macromolecular Chemistry AS CR, vvi, Heyrovsky Sq. 2, 162 06 Prague 6, Czech Republic

**Keywords:** Polyurethane films, Surface structure, AFM microscopy, Surface free energy parameters, Thermal properties, Mechanical properties

## Abstract

In the reaction of 4,4′-methylenebis(phenyl isocyanate), polycaprolactone diol, and *N*-methyldiethanolamine, they were synthesized aqueous dispersions of polyurethane cationomers, from which films were prepared after adding 0–2 wt% graphene. In order to obtain nanocomposites, graphene was previously noncovalent functionalized in tetrahydrofurane in the field of ultrasound. The chemical structure and the morphology of obtained nanocomposites were analyzed by IR spectroscopy, atomic force microscopy (AFM), and differential scanning calorimetry (DSC) microcalorimetry methods. It was found that the presence of graphene results in increased thermal and mechanical strength of received polymer films and contributes to the increase in hydrophobicity of generally hydrophilic coatings prepared from waterborne polyurethane cationomers. Based on received results, possible interactions between graphene and phase structure of polyurethane cationomers were discussed. Relating to the so far described applications of graphene for the modification of polyurethanes, the novelty of this work is the concept of incorporation of graphene particles to polyurethane cationomer chains exclusively through a simple noncovalent functionalization and to investigate the effect of graphene on the properties obtained in this way of thin polyurethane film.

## Introduction

During the past 10 years since the first publication on graphene, a large number of publications regarding the use of the allotropic forms of carbon as a nanofiller in polymer composites were shown [1, 2]. Polymer/graphene coatings compared to the traditional polymer coatings show superior mechanical, thermal, gas barrier, and electrical properties. However, the improvement in the physicochemical properties of these nanocomposites depended on the distribution of graphene layers and polymer bulks as well as the interfacial bonding between graphene layers and polymer matrix. Pristine graphene is not compatible with polymer structure and does not form homogeneous composites [3, 4]. Therefore, numerous methods of chemical functionalization of the graphene were developed from which the greatest attention was paid to the processes of oxidation and synthesis of graphene oxide [5–7]. This is advantageous in terms of mechanical properties but causes a considerable deterioration of electrical properties of the coating.

Unlike graphene, graphene oxide is electrically insulating, which does not allow its application for the production of conducting nanocomposites. So, it is worthwhile to still make new attempts to apply noncovalent methods for graphene modification as a special sonification in organic solvents or in ionic liquids [3].

In recent years, attention is also paid to the possibility of polyurethane material modification by graphene or its functionalized derivatives. New reinforced polyurethane elastomers are being sought in the automotive industry and as varnishes which very useful in the anticorrosive protection of steel and concrete, as well as conducting nanocomposites in electronics [8–10].

In [11], the authors described the possibility of producing graphene-reinforced waterborne nanocomposite coatings by the sol–gel method based on chemically modified graphene and polyurethane anionomers produced from isophorone diisocyanate (IPDI), polyoxypropylene glycol, and dimethylolpropionic acid. In [12, 13], the authors presented the possibility of modifying anionomer polyurethane dispersion produced from IPDI, polycaprolactone diol, and dimethylol butanoic acid by the graphite oxide nanofillers.

Bearing in mind our former achievements concerning the production of waterborne polyurethane cationic dispersion [14], we made attempts to check how the presence of graphene noncovalent functionalized in THF will influence the properties of a polyurethane coating. Previously, we paid particular attention to the reduction of the hydrophilicity of the coatings prepared from polyurethane cationomers [15–17]. It was possible but often has been connected with worsening mechanical properties. For the application of graphene, we are combining here the earlier described possibilities of improvement of the electrical properties of coatings of this type [16]. In the present work, we have limited ourselves to describe a synthesis method and examination of thermal, mechanical, and surface properties of coatings obtained from polyurethane cationomers modified by graphene. Relating to so far described applications of graphene for the modification of waterborne polyurethanes, the novelty of this work is the concept of incorporation of graphene particles to polyurethane cationomer chains exclusively through a simple noncovalent functionalization and to investigate the effect of graphene on the phase structure and physical and mechanical properties of thin films prepared from waterborne cationomer polyurethanes. As shown, the use of graphene indicated the new possibilities of modification of waterborne polyurethane films, although not physically homogeneous nanocomposites were obtained.

## Experimental

### Raw materials and reagents

#### Reagents

**Table:** 

4,4′-Methylenebis(phenyl isocyanate) (MDI), M = 250.25	Aldrich
The isocyanate reagent was used as purchased.	
Poly(ε-caprolactone)diol (PCL), *M*_n_ = 2000	Aldrich
The polyester reagent was dried under vacuum in nitrogen, at 120 °C, for 2–4 h.	
*N*-Methyldiethanolamine (NMDA), *M* = 119.16 $$ \begin{array}{l}\kern7.5em {\mathrm{CH}}_3\hfill \\ {}\kern8em \Big|\hfill \\ {}\mathrm{H}\mathrm{O}\hbox{---} {\mathrm{CH}}_2\hbox{---} {\mathrm{CH}}_2\hbox{---} \mathrm{N}\hbox{---} {\mathrm{CH}}_2\hbox{---} {\mathrm{CH}}_2\hbox{---} \mathrm{O}\mathrm{H}\hfill \end{array} $$	Aldrich
1,6-Hexamethylenediamine (HMDA), *M* = 116.21 H_2_N(CH_2_)_6_NH_2_	Aldrich
Formic acid (HCOOH), 99 %, analytically pure (*M* = 46.03)	POCh S.A., Gliwice, Poland
Dibutyl tin dilaurate (DBTL) [CH_3_(CH_2_)_3_]_2_Sn[OCO(CH_2_)_10_CH_3_]_2_	Huntsman Performance Chemicals
Benzoil chloride	POCh S.A., Gliwice, Poland
Tetrahydrofurane (THF)	POCh S.A., Gliwice, Poland
Analytical reagents: Dibutylamine, diiodomethane, formamide Redistilled water	Aldrich
Graphene Chemical and physical properties are as follows: −Black, crystalline nanopowder 12 nm, company: “Supermarket” −Density 1.8–2.1 g/cm^3^ −Melting point, 3700 °C −The contact area in the solid state, 600 m^2^/g −Contact surface of the dispersion, 1700 m^2^/g	

### Method for the synthesis of polyurethane cationomer coatings with grapheme

Polyurethane cationomers were synthesized in a four-stage polyaddition process, in a glass stand composed of three-necked flask, heating bowl, mechanical agitator, dropping funnel, thermometer, reflux condenser, and nitrogen supply nozzle. At stage 1, urethane-isocyanate prepolymer was synthesized in the reaction of diisocyanate (B) and polyester (A):

1 $$ \mathrm{A}+2\mathrm{B}\to \mathrm{B}\mathrm{A}\mathrm{B} $$

To 7.5 cm^3^ (6.68 g) of THF was introduced 10 g (0.040 m) MDI, and under agitation after reaching a temperature at about 50 °C, two drops of benzoil chloride (inhibitor) and 8.00 g (0.004 m) PCL were added, and then the reaction was continued for 6 h until complete conversion of the polyester, which is when the concentration of –NCO groups amounted to a maximum of 12.5 wt% (determined by using the well-known acidimetry method using dibutylamine). Initially, after introducing PCL, the concentration of NCO groups in the reaction mixture was 13.61 wt% (calculated on the basis of the stoichiometry). Thus, addition of the inhibitor was necessary to limit the side reactions of MDI (present in excess).

In the second stage, suspension of graphene in THF was introduced to the resulting mixture of MDI and BAB prepolymer. Appropriate suspensions (with calculated quantity of graphene (0.024–0.484 g) in THF (7.5 cm^3^)) were obtained in the process of sonication at 50 °C for 30 min in ELMASONIC P ultrasonic baths at a frequency of 80 kHz.

In step 3, respectively 4.147 g (0.0348 m) of NMDA (X) and about 0.012 g of DBTL catalyst were introduced to the reaction system, and then the BAB prepolymer extension reaction was continued at 50 °C for a further 30 min, which shows the following reaction:

2 $$ n\mathrm{B}\mathrm{A}\mathrm{B}+n\mathrm{X}\to -{\left(\mathrm{XBABXB}\right)}_n- $$

The final content of NCO groups in the resulting mixture no longer exceeded 0.25 wt%. Alkylammonium cations were produced by neutralization of tertiary amino groups. For this purpose, to the reaction mixture, 1.60 g (0.0348 m) of the pure HCOOH was introduced.

At stage 4, redistilled water (approximately 36.5 g) with slight amount of NMDA (0.14 g, 0.0012 m) was added under intensive agitation conditions. That stage was intended not only to produce the water dispersion (38–42 wt%), but cationomer chains with the residual –NCO groups were subjected to extension at the same time in the reaction between groups –NCO and water3


or in the reaction between residual groups –NCO and HMDA:4


The resulting dispersion of waterborne polyurethane was degassed in an ultrasonic field.

The reference films were prepared by covering poly(tetrafluoroethylene) (PTFE) or glass plates by the polyurethane cationomer dispersions. Next, the films were placed in a vacuum drier, at 80 °C, over 6 h, and the process of film formation was completed by exposure to ambient air for 10 days. In this way, polymer films containing respectively 0 (PU-0 sample), 0.1 (PU-0.1), 0.5 (PU-0.5), 1 (PU-1), 1.5 (PU-1.5), or 2 wt% (PU-2) of the graphene were received.

### IR spectroscopy

Infrared spectra of the samples were recorded under vacuum at room temperature using a horizontal attenuated total reflectance (ATR) attachment with ZnSe crystal on a Vertex 70v (Bruker) Fourier transform infrared (FTIR) spectrometer in the range of 4000–550 cm^−1^ at a resolution of 2 cm^−1^.

### Atomic force microscopy

Investigation of the topography and heterogeneity relief (PU nanocomposite films were previously freeze-fractured at the temperature of liquid nitrogen) was done by using an atomic force microscope (Dimension Icon, Bruker), equipped with the SSS-NCL probe, Super Sharp SiliconTM-SPM-Sensor (NanoSensorsTM, Switzerland; spring constant 35 N/m, resonant frequency ≈170 kHz). Measurements were performed under ambient conditions using the tapping mode atomic force microscopy (AFM) technique. The scans covered the sizes from 1 × 1 to 50 × 50 μm^2^.

### DSC analysis

Determination of phase transition temperatures of polyurethanes was made by differential scanning calorimetry (DSC) using Mettler Toledo 822 differential calorimeter. Samples of 11–17 mg were tested. Each sample was heated and cooled in a temperature range of −80 to 100 °C, at a rate of 10°/min.

### TG analysis

Thermal gravimetric analysis of the obtained polyurethane foils involved the use of a TGA/DSC1 thermobalance from Mettler Toledo. TG and DTG were derived. The measurements were taken within the temperature range of 25–600 °C, at a constant heating rate of 10°/min, in nitrogen atmosphere. Based on TG thermograms, temperatures corresponding to mass losses of 5, 10, and 50 % were determined.

### Determination of surface free energy for solids

Physical parameters of the surface energy of a solid *γ*_S_ were found on the basis of the Owens-Wendt methods. The first one assumes that the surface free energy *γ*_S,L_ may be presented as a sum of two components [18]:

4 $$ {\gamma}_{\operatorname{S},L}={\gamma}_{\mathrm{S},\mathrm{L}}^d+{\gamma}_{\mathrm{S},\mathrm{L}}^p $$

where

*γ*_S,*L*_^*d*^ is the surface energy connected with dispersive interaction

*γ*_S,L_^*p*^ is the surface energy connected with polar interactions

Equation (4) is generally applicable both to a solid phase, where the subscript *S* is used then, and to a wetting liquid (standard liquid or tested solid material), with the subscript *L*.

The surface free energy (SFE) for solids (S) and for liquids (L) interacting with those solids should satisfy the Owens-Wendt equation:

5 $$ {\gamma}_{\mathrm{L}}\cdot \frac{1+ \cos \varTheta }{2}=\sqrt{\gamma_{\mathrm{S}}^d\cdot {\gamma}_{\mathrm{L}}^d}+\sqrt{\gamma_{\mathrm{S}}^p\cdot {\gamma}_{\mathrm{L}}^p} $$

where *Θ* is the experimentally found contact angle between a liquid drop and a solid surface under investigation. So, wetting angles *Θ* were first measured for the surfaces of polyurethane coatings with the use of two pair model liquids (water–diiodomethane and formamide–diiodomethane) with known parameters *γ*_L_, *γ*_L_^*d*^, and *γ*_L_^*p*^ (Table 1) [19]. Then, Eq. (5) was used to calculate the values *γ*_S_^*p*^ and *γ*_S_^*d*^ for the studied polyurethane films. The values of *γ*_S_ were calculated from Eq. (4). The contact angles *Θ* were measured with the use of the method suggested by Zisman [20], i.e., by an optical goniometer (*Cobrabid Optica*—Warsaw) with a digital camera installed in the axial extension of its lens.

**Table 1:** Surface properties of model measuring liquids [19]

Model measuring liquid	Surface free energy parameters (mJ/m^2^)		
	*γ*_L_	*γ*_L_^*d*^	*γ*_L_^*p*^
Water	72.8	21.8	51
Formamide	58.0	39	19
Diiodomethane	50.8	48.5	2.3

### Mechanical tests

The mechanical strength tests were performed on a testing machine ZWICK type BZ1.0/TH by PN-EN ISO 527–3:1998. Test speed was 100 mm/min, sample width was 10 mm, and gauge length was 50 mm. The following parameters were determined: maximum tensile stress (*σ*_max_), elongation for maximal *σ* (*ε*_m_), tensile strength (*σ*_r_), elongation for tensile strength (*ε*_r_), and Young’s modulus (*E*). It also gives an approximate thickness of the coatings used in the measurements (*b*).

## Results and discussion

Chemical structures of the base polyurethane cationomer synthesized by MDI diisocyanate, which has been used in this work, were the same as described before and confirmed by ^1^H and ^13^C NMR spectroscopy in [21]. In the framework of the present study, only IR spectra were taken.

The key issue was to obtain a good homogeneity of the films obtained from polyurethane cationomers modified by functionalized graphene only by the noncovalent method. Evidence of new interactions between the graphene and the cationomer chain were explored by analyzing the IR spectra. From FTIR analysis, bands related to amine, asymmetric and symmetric methylene, and carbonyl groups were observed at 3300, 2926, and 2863, 1722, and 1710 cm^−1^, respectively (Table 2). The lack of an isocyanate peak at 2250 cm^−1^ and the presence of the amine and carbonyl peaks reveal a complete conversion of monomers to urethane. The two peaks attributed to methylene at 2926 and 2863 cm^−1^ can be attributed to asymmetric CH stretching and symmetric CH stretching, respectively. Carbonyl groups not involved in hydrogen bonding show absorption bands that were observed near 1723 cm^−1^, and carbonyl bands associated with poorly ordered hydrogen bonding were observed at 1710 cm^−1^. Additionally, the increased intensity of the peak around 1711 cm^−1^ in sample with graphene suggests a larger amount of carbonyl group associated with poorly ordered hydrogen bonding. The increased intensity of N–H bands at 3300 cm^−1^, engaged in forming urethane–ester hydrogen bond in the graphene-containing sample, was observed in comparison to unmodified polyurethane. Such polyurethanes synthesized in the reaction of 1,6-hexamethylene diisocyanate with poly(ethylene glycols) (*M* = 2000–8000) and 1,4-butanediol containing 45–77 % hard segment z with dominant crystalline phase therein were characterized by similar IR spectra in the range of the analyzed bands, where in all spectra, a very sharp 3300-cm^−1^ wave band was visible [22]. This can be attributed to the increased degree of phase separation with incorporation of graphene and the reinforcements in the hard segment region.

**Table 2:** Interpretation of FTIR spectra of the PU-0 and PU-1 samples

PU-0	PU-1	Assignment
Wave number (cm^−1^)		
3300	3308	N–H stretching vibrations
3256	3263	
2926	2945	CH_2_ asymmetric
2863	2863	CH_2_ symmetric stretching
1722	1723	Stretching vibrations of carbonyl group (double overlapped vibration bands)
1710	1713	
1597	1597	C–C binding within aromatic ring stretching
1463	1461	
1413	1413	
1533	1532	secondary and tertiary amides
1470	–	C–H
1367	–	C–H wag in CH_2_
1295	–	C–H
1187	1182 (significant intensity decreasing for PU-1.0)	C–O–C bend C–N stretching vibrations
1161	1160	C–O–C bend
1083	1083	C–O stretching vibrations
1060	1064	C–O, C–C stretching, CH_2_ rocking
1045	1047 (lower intensity)	C–O stretching vibrations
933	–	

**Fig. 1:**
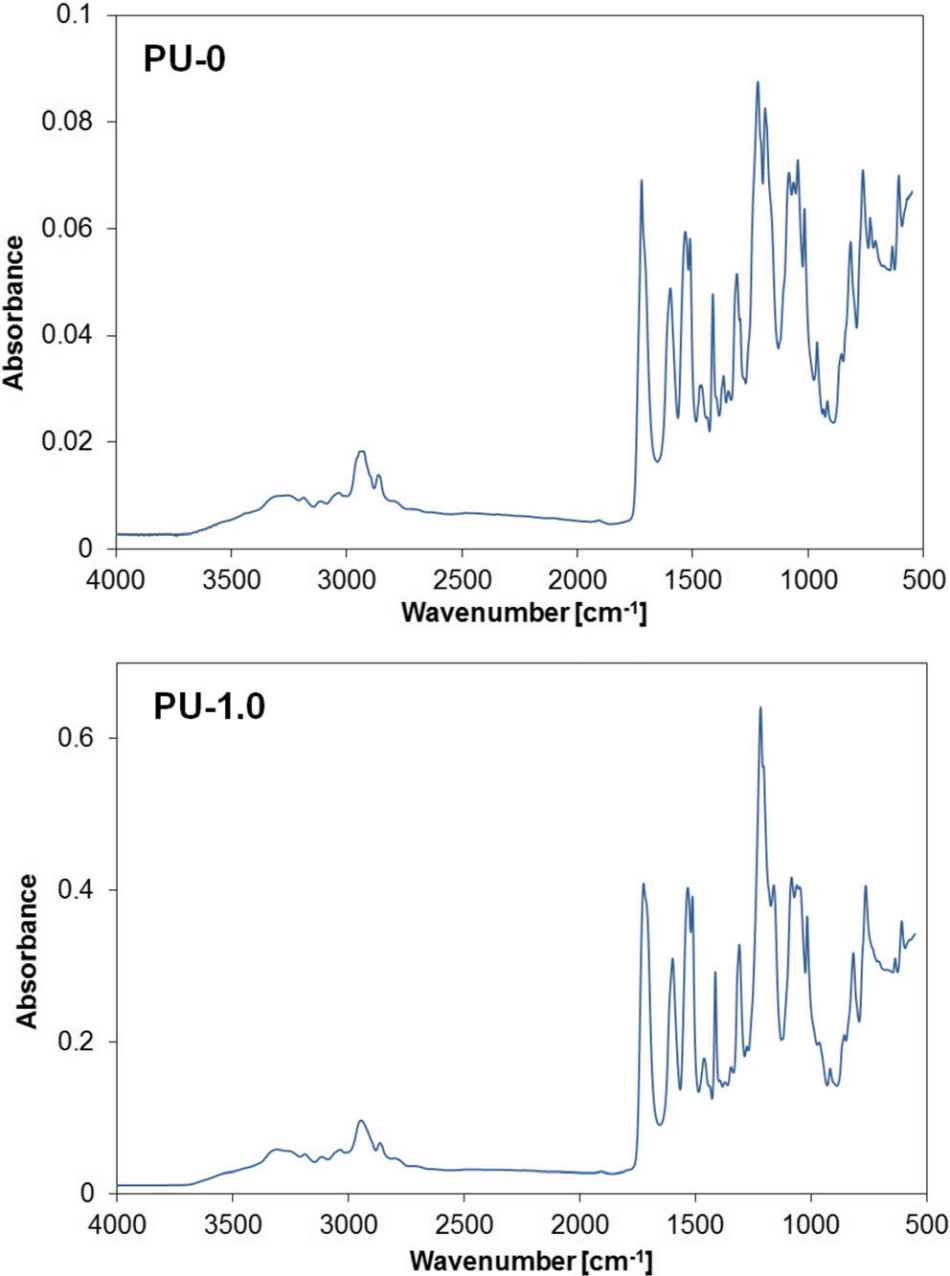
IR spectra of polyurethane cationomer films **a** PU-0 and **b** PU-1

**Fig. 2:**
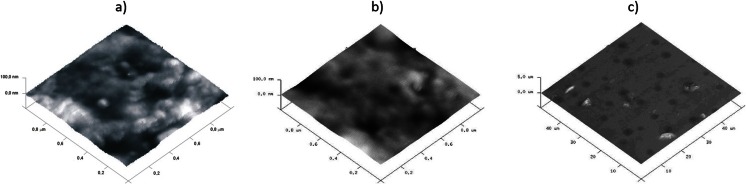
AFM 3D height images of the polyurethane cationomers **a** PU-0 1 × 1 μm, **b** PU-2 1 × 1 μm, and **c** PU-2 50 × 50 μm

**Fig. 3:**
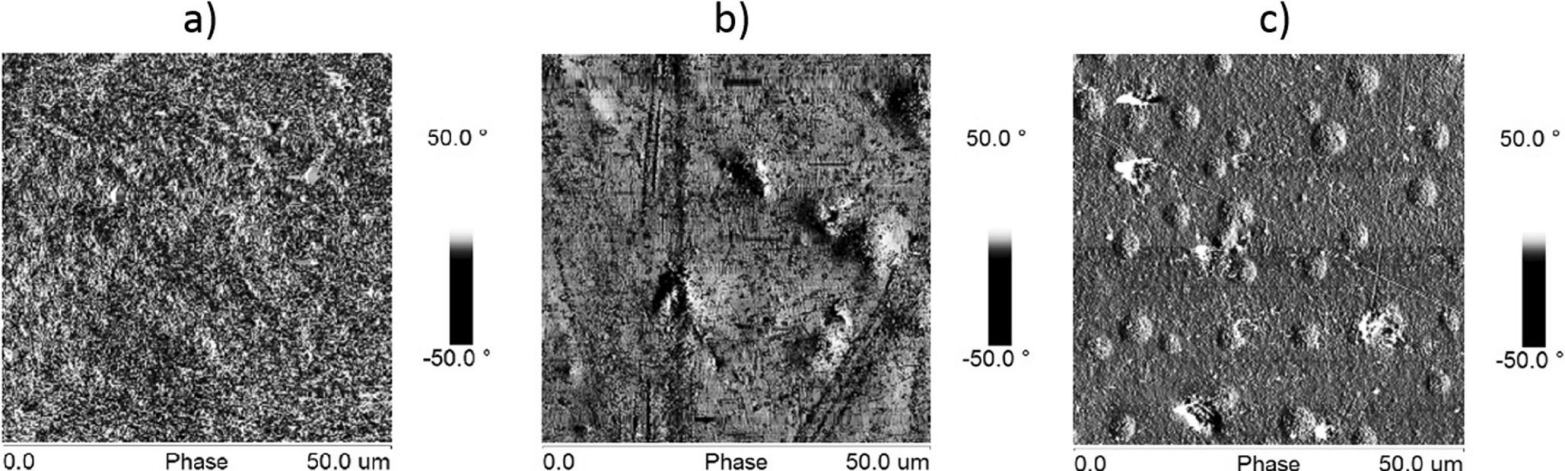
AFM 2D phase images of the polyurethane cationomers **a** PU-0, **b** PU-0.1, and **c** PU-2 for 50 × 50 μm

**Fig. 4:**
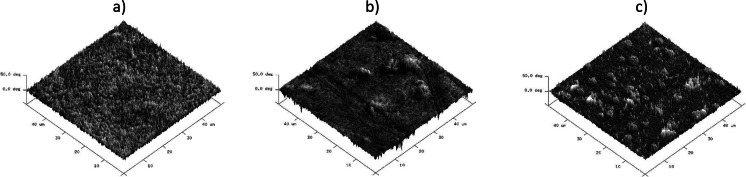
AFM 3D phase images of the polyurethane cationomers **a** PU-0, **b** PU-0.1, and **c** PU-2 for 50 × 50 μm

A detailed comparison of the IR spectra of samples PU-0 and PU-1 (Fig. 1) shows differences in the shape of the bands corresponding to the ether bonds of C–O in the range of 1150–1200 cm^−1^, which may be evidence of the incorporation of the graphene to the noncovalently functionalized PU in the presence of THF. THF as a volatile solvent was removed during drying and seasoning of polymer films. With reference to the above, it is possible to expect that graphene was associated with the polymer matrix, which can in turn be inferred from AFM imaging. It is important that the AFM images were obtained at room temperature immediately after cutting of the sample by a microtome, so they are giving information about the internal structure of material. Already, roughness analysis thus obtained surface made on the basis of the statistical parameters of the sensor height (Table 3) shows that there were no significant differences in surface roughness of samples PU-0 and PU-0.1 and the PU-2 sample surface seems even less rough. This is confirmed by roughness parameters obtained from the sensor height images of different sizes. Comparison of 3D height images (Fig. 2a, b) of the PU and PU-0-2 samples confirms that the formed surface irregularities do not exceed several tens of nanometers. Analysis of the large area (Fig. 2c) of the PU-2 sample, however, indicates the presence of scattered hills with a height of about 1 μm, which indicates the presence of dispersed graphene particles closely surrounded by the polymer matrix. Much more information about the morphology of the phases in the analyzed material was obtained from phase images of the type 2D. AFM image in Fig. 3a shows a “granulation” of the PU-0 structure resulting from phase miscibility typical for polyurethanes composed of rigid segments of urethanes and ureas and flexible segments of polyesters. Rigid segments in cationomers are creating also ionic structures with the participation of alkylammonium groups. However, the phase images of PU-0.1 and PU-2 (Fig. 3b, c) additionally show overlapping heterogeneity due to the presence of graphene particles. Distinct differences in the morphology of the received samples can be seen in the 3D phase images (Fig. 4a–c). On all images presented in Figs. 3 and 4, it can be seen that the graphene particles were well connected with the polymer matrix, although the surface is not homogeneous, which—as demonstrated further—has an effect on the mechanical properties. Graphene particles can be detected on the freeze-fractured surface of PU/graphene nanocomposites by AFM, but without any visible impact on the surface roughness or heterogeneity, as compared with the pure PU matrix. This way, it is possible to assume that the applied noncovalent functionalization of graphene in the presence of THF, made under ultrasound sonication, allowed to obtain nanocomposites, in which graphene was admittedly not very even, but permanently built (anchored) in the polymer matrix. Additional information about the structure of the obtained composites contribute to the DSC analysis.

**Table 3:** Surface properties of the polyurethane cationomer films

Sample no.	The statistical parameters of the surface roughness by AFM height^a^ sensor				Contact angle (°)		Surface free energy (0.001 J/m^2^)		
	Surface area (μm^2^)	*R*_a_ (nm)	*R*_q_ (nm)	*R*_max_ (nm)	Water	Diiodomethane	*γ*_S_	*γ*_S_^*d*^	*γ*_S_^*p*^
PU-0	1	3.43	4.41	27.6	72.4	26.4	45.3	40.5	4.8
	100	17.6	22.0	159					
	2500	28.4	35.2	361					
PU-0.1	1	2.63	3.43	23.1	76.9	32.9	42.3	39.2	3.1
	100	66.3	94.2	572					
	2500	65.7	94.4	887					
PU-0.5	–	–	–	–	80.1	35.0	41.4	38.9	2.5
PU-1.0	–	–	–	–	81.0	40.8	38.7	35.8	2.9
PU-1.5	–	–	–	–	81.1	39.2	39.4	36.8	2.6
PU-2	1	2.16	2.77	20.6	84.8	41.3	38.4	36.7	1.7
	100	5.00	7.56	174					
	2500	15.4	32.0	657					

*R*_a_ (mean roughness) is the mean value of the surface relative to the center place. *R*_q_ (*R*_ms_) is the standard deviation of the *Z* values within the given area. *R*_max_ (max height) is the difference in height between the highest and lowest points on the surface relative to the mean plane. Mean is the average of all *Z* values within the enclosed area

^a^Surface area: the total area of examined sample surface (the three-dimensional area of a given region expressed as the sum of the area of all the triangles formed by three adjacent data points)

**Fig. 5:**
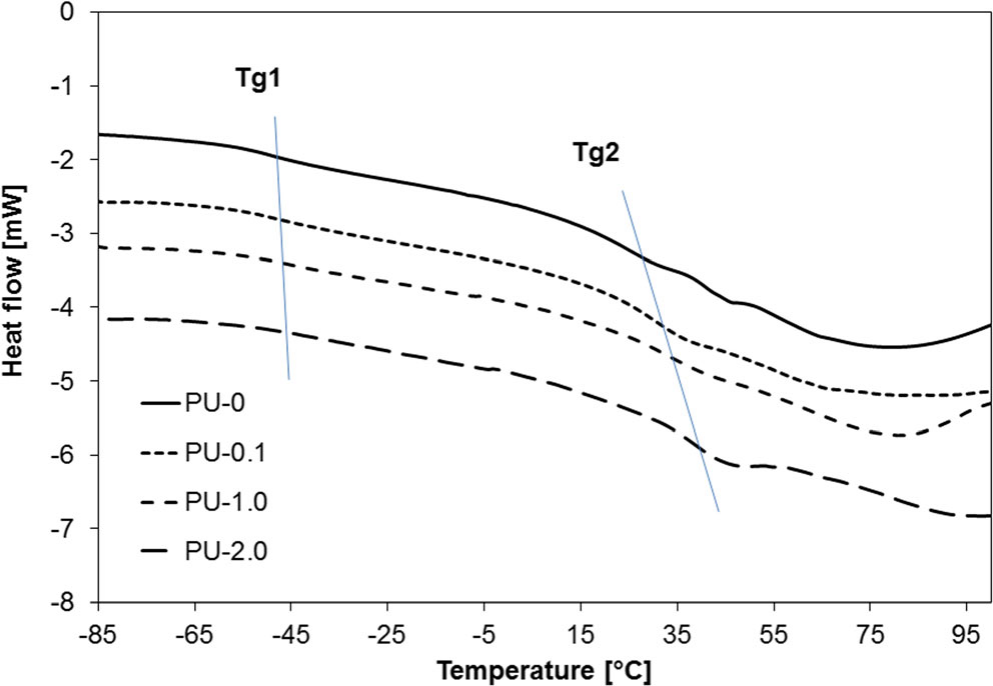
DSC thermograms of the PU and PU/graphene nanocomposites

**Fig. 6:**
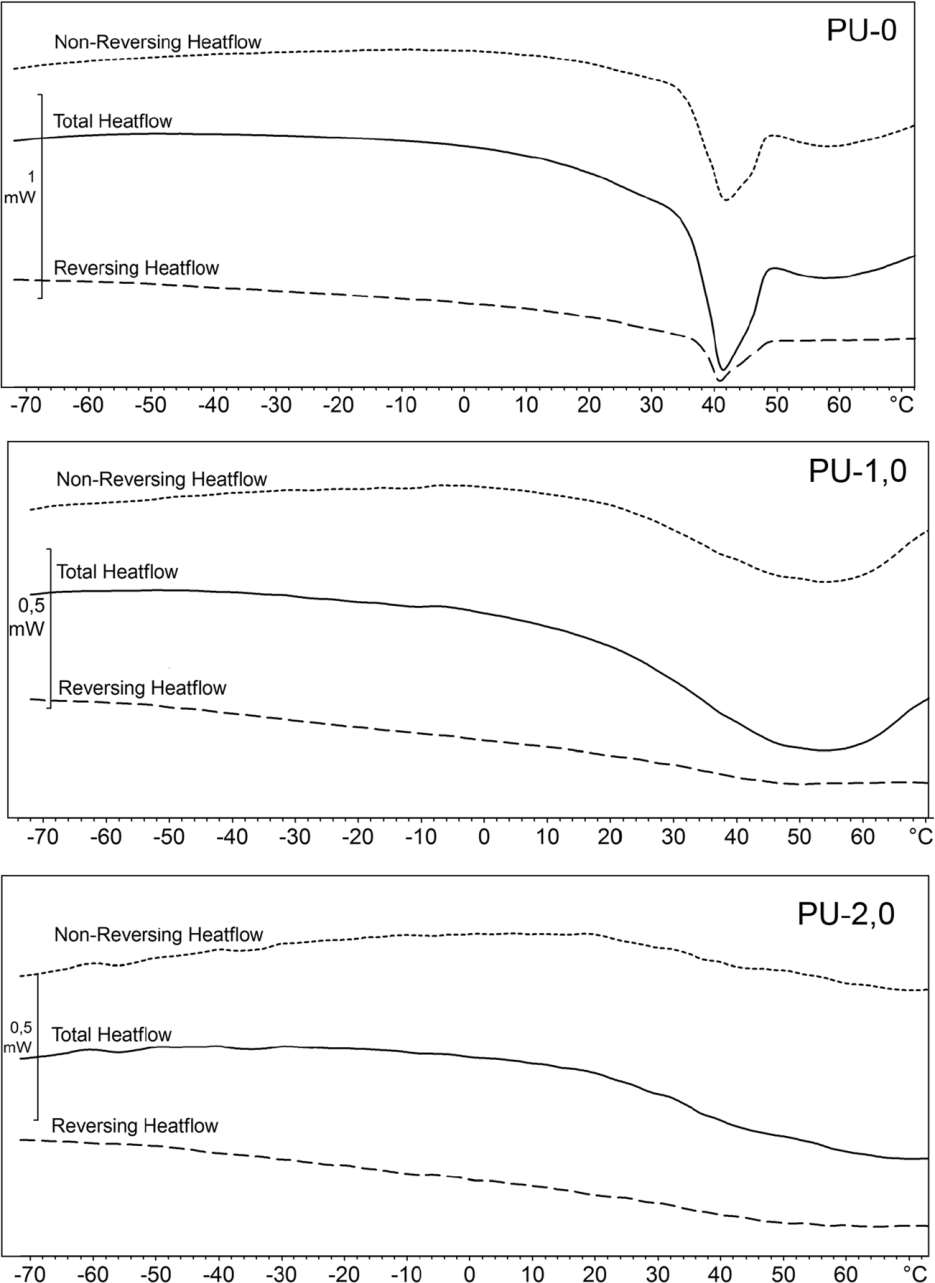
TOPEM DSC thermograms of the PU and PU/graphene nanocomposites

As it can be seen from Fig. 5 and Table 4, glass transition of soft segments is in the range from −50 to −43 °C and glass transition of hard segments occurs in the temperature range of 23–39 °C. The obtained results are typical for MDI-based polyurethanes obtained from both polyethers and polyesters [23]. In [21], it is shown that in the case of polyurethane cationomers, *T*_g2_ increases with the rise in the amount of built-in alkylammonium cations, whose participation in the here-synthesized cationomers did not change and was about 2.0 %. It should be noted that the introduction of graphene leads to an increase in the glass temperature of soft segments (*T*_g1_) only from −49.7 to −43.4 °C, while for hard segments, a significant increase of glass temperature (*T*_g2_) from 23.9 to 38.6 °C (as well as an increase in delta *C*_p_) was observed. The higher *T*_g1_ can reflect the changes in long-range segmental mobility in soft segments due to the introduction of graphene. It enhances the rigidity of the soft segments and results in a slight increase in phase transition temperature of PU soft segments. As shown in Table 2, delta *C*_p_ for hard segments increases and, in consequence, the segmental mobility is significantly reduced in samples with graphene, indicating that chain immobilization occurs when they are intercalated between graphene layers. Similar results were observed for PU/organomodified montmorillonite where PU chain mobility was constricted by MMT galleries [24]. This behavior was also compared to that of semi-crystalline polymer where a rigid amorphous fraction is typically observed and chain movement is restricted by surrounding lamellar crystals [25]. In our system, graphene layers can act as lamellar crystals restricting polymer chain movements. Moreover, a small peak at 45 °C for PU without graphene was observed and it can be attributed to the melting process of PCL-based soft segments [26]. For samples modified with graphene, no melting peaks were detected that can indicate that graphene hinders crystallization of soft segments. This phenomenon can be attributed to the reduced chain mobility between graphene layers. These results were also confirmed in TOPEM DSC results (Fig. 6) where the melting process was observed only for unmodified PU. The observed differences indicate a change not only in the surface morphology of obtained nanocomposites but reaching the interior of it with permanently built-in graphene.

**Table 4:** Glass transition parameters by DSC and TOPEM DSC methods

Sample no.	Glass transition of soft segments (°C)			Glass transition of hard segments, °C		
	*T*_g1inflection point_ (conventional DSC)	*T*_g1inflection point_ (TOPEM DSC)	Δ_1_ *C*_p_ (J/g deg) (conventional DSC)	*T*_g2inflect_ (conventional DSC)	*T*_g2inflection point_ (TOPEM DSC)	Δ_2_ *C*_p_ (J/g deg) (conventional DSC)
PU-0	−49.7	−43.5	0.178	23.9	26.0	0.297
PU-0.1	−49.4	–	0.181	30.9	–	0.377
PU-1.0	−45.2	−43.6	0.127	32.3	32.8	0.310
PU-1.5	−47.9	–	0.180	37.3	–	0.442
PU-2	−43.4	−43.8	0.147	38.6	38.9	0.329

**Fig. 7:**
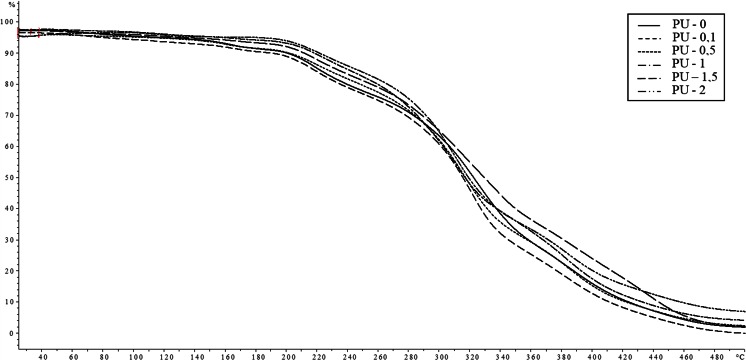
TG thermograms of the synthesized nanocomposites

Stiffening of the structure by introducing a graphene clearly resulted in increased heat resistance and mechanical strength of the obtained nanocomposites. With this, the pronounced increase in the thermal resistance—as seen from the TG thermograms shown in Fig. 7 and in the results listed in Table 5—occurs only after 1 % content of graphene.

**Table 5:** Thermal and mechanical properties the polyurethane cationomer films

Sample no.	Thermal properties					Mechanical properties					
	*T*_5%_	*T*_10%_	*T*_50%_	*T*_max_	Ash (%)	*b* (mm)	*σ*_max_ (MPa)	*ε*_m_ (%)	*σ*_*r*_ (MPa)	*ε*_r_ (%)	*E* (MPa)
PU-0	115	206	317	497	4.67	0.40	10.65	10	5.51	145	162
PU-0.1	115	194	317	495	5.40	0.18	68.09	470	68.07	471	334
PU-0.5	116	204	318	499	6.57	0.14	7.91	10	5.82	130	85
PU-1.0	172	220	318	495	6.80	0.12	38.66	510	38.65	508	267
PU-1.5	180	212	320	491	7.50	0.21	13.35	461	13.17	462	194
PU-2	182	223	323	496	11.24	0.23	58.57	508	58.35	509	383

**Fig. 8:**
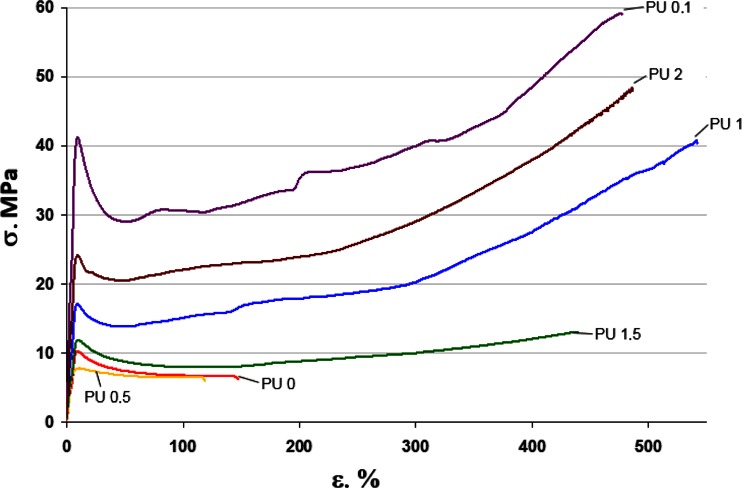
*σ*–*ε* mechanical curves of the polyurethane films

The character of changes of registered curves is presented on Fig. 8. However, the mechanical parameters read out from these curves are demonstrated in Table 5. Omitting the PU-0.5 sample which did not achieve reliable results (as similarly as in case of DSC analyses), one can see the distinct increase in the mechanical strength with the rise in the amount of graphene, although a distinct dependence on the quantity of this nanofiller was not observed. Received covers are flexible with relatively large values of elongation (over 400 %) and the Young’s modulus in the range of 200–400 MPa. Observed ambiguous dependence of appointed mechanical parameters on the amount of graphene can be explained by the heterogeneity of the phase-obtained nanocomposites, as shown by AFM analysis.

As shown in our earlier studies [15], the chemical structure and phase construction of cationomers significantly influence the surface free energy parameters of received polymer coatings. Analyses performed at this work showed additionally that the presence of graphene increases the hydrophobicity of the obtained nanocomposites. From the data presented in Table 3, results that produced polymer films are characterized with higher values of contact angles for water and diiodomethane along with the rise in the amount of graphene, which results in lowering the surface free energy from 45 to 38 mJ/m^2^, which is a significant value. Lowering of the surface free energy is observed in both values of the dispersion component *γ*_S_^*d*^ and—with a much lower value—the polar component *γ*_S_^*p*^. This result is seemingly surprising enough in the light of previous results concerning DSC and AFM analyses, which indicated the presence of additional interactions within the rigid segments. Here, it is necessary to consider that the measurements of contact angles in contrast to the AFM analysis were performed on real coatings and thus they do not characterize the internal structure of the material as the internal surface analysis. It is worth noting that, as presented in [15], a mathematical model allowing for the estimation of the surface free energy parameters based on the chemical structure of the chains of cationomers creates opportunities for a broader consideration of SFE changes of coatings additionally modified by graphene. An observed increase in the hydrophobicity of produced films results explicitly from the presence of apolar graphene particles, and for a number of applications, it may be preferred. The work [27] indicated that the hydrophobic films from waterborne polyurethane ionomers may exhibit excellent long-term barrier properties beneficial for their application as protective coatings. The additional presence of graphene may here be an advantage in applications requiring increased electrical conductivity.

## Conclusions

It has been shown that the multi-step method of synthesis of the polyurethane cationomer allows already at the stage of synthesis of the isocyanate prepolymer to introduce graphene in the form of a suspension in THF. Advantageous features of graphene for its homogenization in polyurethane mixture were obtained during its noncovalent functionalization in THF—a solvent often used in the preparation of polyurethane ionomers. The possibility of chemical influences of graphene with THF and with rigid segments of chains of synthesized cationomers provides subtle changes in the IR spectra as well as in the AFM and DSC analyses. The influence of graphene on the nature of interactions within the rigid segments indicates the observed increase of *T*_g2_ at about 15° in comparison with an unmodified cationomer. Crucial for explaining the homogeneity of morphology of obtained nanocomposites are the results of AFM analyses regarding the interior of the material. The surfaces of the tested samples showed a relatively small increase of roughness with increasing amounts of graphene. However, the surface morphology was complex; an inequality maximum of up to 1 μm and a phase heterogeneity especially in graphene particles were observed. Analysis of phase images indicates that graphene was permanently associated with the polymer matrix. The consequence of the existing heterogeneity of nanocomposite structures was weaker than the expected mechanical properties of the obtained films, although graphene greatly improved the mechanical strength of the modified cationomer coatings, so that its introduction at 0.1 wt% resulted in an increase in Young’s modulus of up to about 400 MPa and a relative elongation of up to 400 %. There was also a distinct improvement in thermal resistance of the obtained composites and a regular increase in the hydrophobicity of the coatings with increasing amounts of graphene, which result in the polar material with average surface free energy of 38 mJ/m^2^, while the output coating was characterized by an SFE value equal to 45 mJ/m^2^. In summary, it has been shown that even a relatively simple noncovalent functionalization of graphene may be sufficient for the preparation of polymer films produced from waterborne polyurethane cationomers.
